# Editorial: Recent insights on thermophilic anaerobic bacteria

**DOI:** 10.3389/fmicb.2026.1833446

**Published:** 2026-04-08

**Authors:** Johann Orlygsson, Sean M. Scully, Ed W. J. van Niel

**Affiliations:** 1 Faculty of Natural Resource Sciences, University of Akureyri, Akureyri, Iceland; 2 Department of Applied Biotechnology, Lund University, Lund, Sweden

**Keywords:** anaerobic, fine chemicals, genetic engineering, thermophilic, thermozymes

Thermophilic anaerobic bacteria (TAB) find their niche in oxygen-free environments at 50–90 °C, such as hydrothermal vents and petroleum reservoirs. These bacteria can be classified according to their optimal growth temperatures: (moderate) thermophiles (50–64 °C), extreme thermophiles (65–80 °C), and hyperthermophiles (>80 °C). TAB are metabolically versatile, fermenting various carbohydrates and degrade complex biomass structures and often have higher metabolic rates than their mesophile counterparts, making them of interest for advancing industrial biotechnology. TAB have been broadly applied to challenges in biotechnology such as, but certainly not limited to, producing biofuels, fine chemicals, bioremediation, and wastewater treatment. Their metabolism relies on highly stable, heat-resistant enzymes (thermozymes) that can be exploited for converting organic waste into value-added products. New promising anaerobic thermophilic species are isolated and characterized on a regular basis.

Several new genetic tools have been developed in the last 5 years, including CRISPR-Cas9 editing systems, for many TAB to improve their performance, make them more robust to extreme environmental conditions and produce new chemicals. However, the synthetic biology toolbox for TAB remains restricted because more development is required. TAB are regarded as a vast and valuable reservoir of thermozymes. Recently characterized thermozymes include hydrolases with broad utility including xylanases, cellulases, amylases, lipases, and proteases in addition to the classic example of thermostable DNA polymerases that have enabled modern biotechnology.

This Research Topic consists of six publications, including four original research articles, one review article, and a minireview that each address particular thermophiles producing products of interest or thermozymes. Below, we discuss these contributions and their broader implications of this work in the context of exploring TABs and their utility.

Tapping into the diversity of thermophilic organisms as source of producing a range of useful molecules using a variety of culture-based and culture-independent techniques present a range of approaches. Zhong et al. present in a review the properties of the “unclassified” family of Thermoactinomycetaceae, a promising microbial resource of novel specific enzymes for the synthesis of added-value products and novel secondary metabolites, and for efficient biomass conversion.

Beyond using lignocellulosic biomass as a feedstock for producing useful biomolecules, the use of seaweeds is an area that is of tremendous interest. Ingvadottir et al. describe the fermentation of L-rhamnose to 1,2-propanediol by the moderate thermophilic *Clostridium* strain AK1. The rhamnose was obtained from hydrolysis of the macroalgae *Ulva lactuca* and a maximum yield of 79% was reached although challenges with completely utilizing the available carbohydrates remain. Regardless, the study reveals the potential of the macroalgae as a feedstock for production of added-value compounds.

Expanding upon the knowledge of thermozymes with potential applications in biomass deconstruction, Huang et al. isolated a novel β-glucosidase, which also possesses β-galactosidase activity, from a hot spring in the Rehai area, Tenchong County in Southwest China. It has been identified to belong to the GH1 family and its kinetics was determined. It is a promising thermozyme for industrial applications, such as bio-ethanol production, and cellulose hydrolysis due to its remarkable enzymatic properties particularly due to its insensitivity to glucose inhibition.

Galindo et al. describe the heterologous expression of a robust enzymatic reporter system from hyperthermophilic archaeal galactosidases in the extreme thermophilic bacterium *Anaerocellum bescii*. These reporter systems possess a high signal-to-noise ratio and are handled easily, which make them favorable genetic tools to be used for industrial biotechnology.

Developing genetic tools for the manipulation of TABs remains a topic of great importance. In a minireview, Bourgade and Islam go into recent developments in metabolic engineering of thermophilic acetogens. These bacteria are recognized as potential microbial cell factories converting C1 substrates into acetate and other organic compounds.

Finally, Ye et al. report on a nisin-controlled expression vector in *Streptococcus thermophilus* to enhance expression efficiency but this bacterium has been used as a microbial cell factory for producing food ingredients and high value metabolites and its importance has increased recently because of its host capabilities for genetic engineering.

We acknowledge the talented group of researchers who have contributed to this volume. We started this Research Topic, to gather diverse research articles on the various biotechnological aspects of anaerobic, thermophilic bacteria. We hope that this Research Topic will initiate new studies in the exciting area of bacteria from warm, oxygen-free habitats.

